# Mechanisms and Therapeutic Strategies to Overcome Immune Checkpoint Inhibitor Resistance in Melanoma, Head and Neck, and Triple-Negative Breast Cancers

**DOI:** 10.33696/immunology.7.235

**Published:** 2025

**Authors:** Iryna Voloshyna, Apoorvi Tyagi, Stanzin Idga, Nicole Wang, Tazrif Amin, Madonna Hanna, Adil Mukhtar, Francesca Torres, Farah Kabir, Dominic Florian, Chloe Wang, Yury Patskovsky, Michelle Krogsgaard

**Affiliations:** 1 Laura and Isaac Perlmutter Cancer Center at NYU Langone Health, New York, NY, USA; 2 Department of Pathology, NYU Grossman School of Medicine, New York, NY, USA

**Keywords:** Immune checkpoint inhibitors, Immunotherapy resistance, Melanoma, Head and neck squamous cell carcinoma, Triple-negative breast cancer, Tumor microenvironment, Tumor mutation burden, Neoantigens, Oncogenic pathways, Antigen presentation

## Abstract

Immunotherapy, particularly immune checkpoint inhibitors (ICIs), has revolutionized cancer treatment by harnessing the host immune system to target malignancies. Melanoma, head and neck squamous cell carcinoma (HNSCC), and triple-negative breast cancer (TNBC) were among the first solid tumors to gain regulatory approval for ICIs due to their immunogenicity and unmet clinical needs. Melanoma exemplifies the success of ICI therapy, with durable responses driven by its high mutation burden and neoantigen landscape, yet both primary and acquired resistance remain major challenges. In contrast, HNSCC demonstrates clinically meaningful but modest responses in the context of a highly immunosuppressive tumor microenvironment, while TNBC derives limited benefit from ICI, often requiring combination strategies to achieve efficacy. Resistance to ICIs arises from complex tumor-intrinsic, microenvironmental, and systemic mechanisms that collectively undermine effective anti-tumor immunity. This review highlights both shared and cancer-specific mechanisms of ICI resistance across melanoma, TNBC and HNSCC. We also discuss emerging strategies, including combination therapies, neoantigen-based vaccines, adoptive T cell therapies, and precision oncology approaches, to overcome resistance and improve clinical outcomes. Together, these insights provide a framework for optimizing immunotherapy and advance durable benefit in these challenging malignancies.

## Introduction

Immunotherapy has fundamentally transformed modern oncology, offering durable clinical responses and opening new therapeutic avenues for a wide range of malignancies. By harnessing the host immune system to target and eliminate cancer cells, therapies such as immune checkpoint inhibitors (ICIs) have achieved breakthroughs in cancers previously considered treatment-refractory [1,2]. The approval of ipilimumab, a cytotoxic T-lymphocyte-associated protein 4 (CTLA-4) antibody, for advanced cutaneous melanoma in 2011 marked the dawn of a new era in cancer treatment [3]. Subsequent regulatory approvals of programmed death-1 (PD-1) and its ligand (PD-L1) inhibitors have rapidly expanded the impact of ICIs to multiple solid tumors, including head and neck squamous cell carcinoma (HNSCC) and triple-negative breast cancer (TNBC) [2,4–6]. These cancers remain at the forefront of clinical and translational immuno-oncology.

Melanoma, HNSCC, and TNBC exemplify both the successes and limitations of current immunotherapeutic strategies. In melanoma, historically dismal outcomes with chemotherapy or interleukin-2 (IL-2) therapy have been replaced by unprecedented long-term survival in a subset of patients, driven by its high tumor mutational burden, abundant neoantigen repertoire, and a tumor microenvironment conducive to T-cell infiltration [7–9]. Despite these advances, most patients ultimately develop primary or acquired resistance (relapse after initial benefit) [10–12]. Similarly, ICIs have reshaped the therapeutic landscape in HNSCC, a cancer often associated with oncogenic viral infection, tobacco and alcohol exposure, and often marked by a profoundly immunosuppressive tumor microenvironment (TME) [13, 14]. Although PD-1 blockade has provided meaningful improvements in recurrent or metastatic disease, only a subset of patients experiences durable benefit, reflecting resistance mechanisms driven by tumor heterogeneity, immune exclusion, and adaptive immunosuppression [14]. TNBC, the most aggressive breast cancer subtype characterized by the absence of estrogen receptor (ER), progesterone receptor (PR), and human epidermal growth factor receptor 2 (HER2), has likewise benefited from ICI-based combinations. The addition of pembrolizumab to chemotherapy has improved outcomes in both early-stage and metastatic settings [15–17]. Nevertheless, durable responses remain uncommon, underscoring the need to better understand tumor-immune escape and to identify strategies that extend therapeutic benefit.

Resistance across these cancers, whether primary (non-response) or acquired (relapses following initial response), emerges from a multifaceted interplay of tumor-intrinsic factors (e.g., antigen-presentation loss, signaling pathway alterations) and microenvironmental barriers (e.g. immunosuppressive cells, stromal remodeling) and systemic host-related constraints (e.g. metabolism, microbiome) that collectively blunt effective antitumor immunity [18–20].

This review examines melanoma, HNSCC, and TNBC as model immunogenic epithelial cancers responsive to ICI. We highlight both convergent and cancer-specific resistance mechanisms, explore their clinical implications, and discuss emerging therapeutic strategies—including rational ICI combinations, neoantigen-targeted vaccines, adoptive T-cell therapies, and precision-based patient selection. By integrating current insights, we aim to provide a framework for overcoming resistance and optimizing immunotherapy outcomes for these difficult-to-treat malignancies.

## Current Clinical Landscape of Immunotherapy and ICI Resistance

Over the past decade, immune checkpoint blockade has become a standard of care across multiple malignancies. ICIs function by removing the inhibitory signals on T-cell activity, most prominently through pathways involving CTLA-4 (CD152) [21], PD-1 (CD279)/PD-L1 (CD274) [22]. While the number of newly identified immune checkpoint molecules is rapidly expanding, the clinically approved portfolio of ICIs remains limited, reflecting the complex biology of checkpoint regulation [23] and the highly variable efficacy of these agents across tumor types [24]. This variability is strongly influenced by tumor-intrinsic biology and the characteristics of the tumor microenvironment (TME) (Table 1).

### Melanoma

Among solid tumors, cutaneous melanoma shows the highest responsiveness to ICIs, in contrast to acral and uveal melanomas, which are generally refractory to these therapies [25–28] (Table 1). Prior to ICIs, metastatic disease was associated with a median survival of less than one year, with minimal benefit from chemotherapy or high-dose interleukin-2 [29]. The introduction of anti-CTLA-4 inhibitor (ipilimumab) and PD-1 inhibitors (nivolumab, pembrolizumab) transformed outcomes for advanced disease [25] (Table 2). Landmark clinical trials, including MDX010–20 [30], KEYNOTE-006 [31], and CheckMate-067 [32], not only demonstrated objective response rates (ORRs) of ~40–45% but also achieved unprecedented long-term survival, with > 40% overall survival (OS) at 6.5 years in some cohorts [33]. Pembrolizumab has shown particularly strong activity in desmoplastic melanoma, with ORRs approaching 89%, high rates of pathological complete response (pCR), and extended disease-free survival [34]. Combination regimens (ipilimumab plus nivolumab) have further improved progression-free survival (PFS) compared with monotherapy. For patients with BRAF-mutant melanoma, the Phase III DREAMseq trial established the sequencing of targeted therapy (dabrafenib/trametinib) after combined ICI (nivolumab/ipilimumab) as the preferred treatment strategy (Table 2), demonstrating a 30% improvement in OS and a threefold improvement in PFS at 5 years compared with either therapy alone [35].

Despite these advances, resistance to ICI remains a significant clinical challenge, presenting either as primary non-response or acquired relapse. Approximately 55% of melanoma patients have primary resistance to PD-1 inhibitors, 40% to CTLA-4+PD-1 combination therapy, and 25% of initial PD-1 responders acquire resistance within two years [36]. Mechanistic drivers of resistance include loss of antigen-presentation [37], defects in interferon-γ (IFN-γ) signaling [38,39], activation of the WNT/β-catenin pathway [40–42], upregulation of compensatory inhibitory checkpoints [43], and recruitment of immunosuppressive myeloid cells [44] (Table 1). Many of these mechanisms are now being targeted with rational ICI-based combinations. For example, melanoma’s high mutational and neoantigen load made it the first tumor type to be evaluated in clinical trials of personalized neoantigen mRNA vaccine. The Phase IIb KEYNOTE-942 trial (mRNA-4157/V940, Merck and Moderna) [45] demonstrated that adding a personalized vaccine to pembrolizumab significantly improved recurrence-free survival (79% vs. 62%) and distant metastasis-free survival (92% vs. 77%) at 18 months compared with pembrolizumab alone (Table 1) [45]. The recent approval of anti-lymphocyte activation gene-3 (*LAG-3*) therapy (relatlimab plus nivolumab) further illustrates how rational ICI combinations are expanding the potential for durable response [46].

### HNSCC

HNSCC is biologically heterogeneous, influenced by risk factors such as tobacco, alcohol, and human papillomavirus (HPV) infection, which significantly shape the tumor immune landscape [94]. Historically, platinum-based chemotherapy has served as the backbone of first-line therapy for recurrent or metastatic HNSCC. HPV-positive (HPV+) tumors are generally more inflamed and responsive to ICIs, whereas HPV-negative (HPV-) tumors often exhibit immune exclusion and profound immunosuppression [95].

The clinical efficacy of PD-1 blockade in platinum-refractory HNSCC was established through the CheckMate-141 (nivolumab) and KEYNOTE-040 (pembrolizumab) trials, confirming ICIs as a standard of care in this setting [68] (Table 2). The role of PD-1 inhibitors was later expanded to the first-line therapy in KEYNOTE-048, where pembrolizumab improved outcomes both as monotherapy for PD-L1-positive tumors and in combination with platinum/5-FU for all patients [66]. Despite durable benefits in a subset of patients, objective response rates remain modest (~15–20%) [96–98] (Table 1). KEYNOTE-012 [99] and KEYNOTE-055 [100] confirmed comparable efficacy for PD-1 blockade between HPV+ and HPV- populations in recurrent/metastatic HNSCC. Similarly, CheckMate-141 demonstrated improved response rates (13.3% vs. 5.8%) and OS in 361 platinum-refractory HNSCC patients treated with nivolumab, with no significant difference between HPV+ and HPV- status [101] (Table 2). In contrast, a PD-L1 inhibitor, durvalumab, has not demonstrated benefit in this setting. Phase III trials of durvalumab alone or in combination with tremelimumab (a CTLA-4 inhibitor) failed to improve OS compared with chemotherapy, limiting its clinical role in recurrent/metastatic HNSCC [102,103].

Resistance in HNSCC is multifactorial (Table 1), including loss of MHC I expression, defective IFN-γ signaling, T-cell exclusion, and expansion of regulatory T cells (Treg) and myeloid-derived suppressor cells (MDSCs) within the TME [96,104,105]. Like melanoma and TNBC, many HNSCC tumors upregulate PD-L1 in response to IFN-γ [106], and alterations in the phosphoinositide-3-kinase (PI3K) - phosphatase and tensin homolog (PTEN) pathway contribute further to tumor evasion due to the developed dysfunction of immune cells [24,107]. HNSCC exhibits unique features as HPV+ tumors evade immunity via viral oncoproteins E6/E7 [107], displaying high T-cell infiltration but increased Tregs and CTLA-4 expression [22,23]. Tobacco-associated tumors have reduced immune infiltration despite high mutational burden, consistent with poorer outcomes [109,110]. Additionally, HNSCC displays distinctive natural killer (NK)-cell biology, characterized by abundant CD56^dim^ NK cells [13,111]. The NK activity is suppressed through killer cell immunoglobulin-like receptor (KIR) signaling and can be influenced by HPV status [13] (Table 1). Emerging treatment strategies for HNSCC include dual checkpoint blockade (PD-1 plus CTLA-4 or LAG-3) and combinations with radiation, vaccines, or targeted therapies aimed at reprogramming the immune TME [112,113].

### TNBC

TNBC, defined by the absence of ER, PR, and HER2 expression, is associated with poor prognosis and limited targeted treatment options. Early-phase studies demonstrated modest efficacy of PD-1 or PD-L1 inhibitors as monotherapy (~5–20% ORR) in TNBC patients [82,114] (Table 2). The Phase I JAVELIN trial of avelumab (targeting anti-PD-L1) reported ORRs of 44.4% in PD-L1-high versus 2.6% in PD-L1-low TNBC patients [84]. Single-agent efficacy remains limited, with progression driven by intrinsic resistance mechanisms such as immune exclusion and adaptive resistance pathways [84]. TNBC is typically characterized by low TMB, limited tumor-infiltrating lymphocytes (TILs), and strong stromal barriers, making it less responsive to ICIs than melanoma [115] (Table 1). Approximately 20–30% of TNBC tumors express PD-L1, often correlating with higher TIL infiltration and higher histological grade [116], providing an additional target for ICI.

Combination strategies have proven more effective. Meta-analyses show that ICIs combined with anthracyclines and taxanes significantly increase pCR (64.8%) in early TNBC while reducing toxicity compared to platinum chemotherapy [117]. Combining anthracycline and taxane chemotherapy with durvalumab as adjuvant therapy can improve the prognosis of early TNBC [118]. The IMpassion130 trial demonstrated that atezolizumab plus nab-paclitaxel improved PFS and OS in PD-L1-positive metastatic TNBC, although IMpassion131 failed to replicate these benefits (Table 2) [117]. KEYNOTE-35 trial found that pembrolizumab plus chemotherapy extended OS in PD-L1–high (CPS≥10) metastatic TNBC patients (median OS of 23.0 vs. 16.1 months) [77]. In early disease, KEYNOTE-522 showed that pembrolizumab plus neoadjuvant chemotherapy improved pCR (64.8% vs. 51.2%) and event-free survival [119].

Despite these advances, TNBC remains largely ICI-refractory [120] (Table 1). Shared resistance mechanisms include immune exclusion, defective antigen presentation, and suppressive myeloid infiltration, but TNBC exhibits certain distinct features: low TMB, absence of pre-existing tumor-specific immunity, stromal-mediated T-cell exclusion, and macrophage-driven suppression. High genomic instability is another hallmark of TNBC [20,121,122]. While challenging, it also presents opportunities for targeted interventions, such as poly (ADP-ribose) polymerase (PARP) and Protein Kinase B (AKT) inhibitors, to enhance response rates [123]. TNBC tumors often display immune-excluded phenotypes, characterized by a lack of immune cell infiltration into the tumor parenchyma due to a dense stromal matrix and TGF-beta-induced fibrosis acting as physical barriers [121,122].

## Mechanisms of Resistance to ICI Therapy

**Figure 1.**
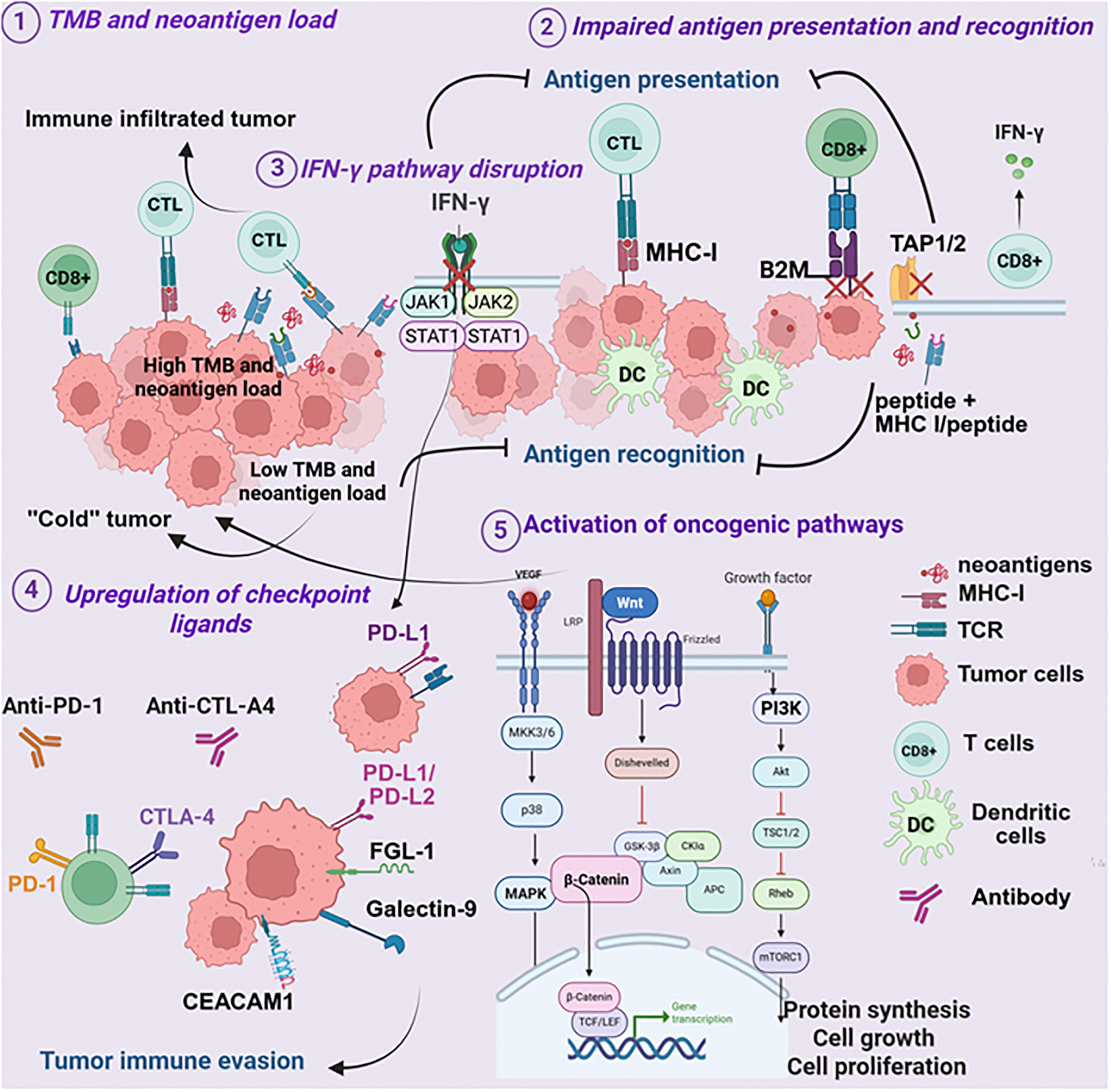
Tumor-intrinsic mechanisms of resistance to ICIs. **(1)** Variations in tumor mutational burden (TMB) and neoantigen load limit tumor recognition and reduce immune cell infiltration; **(2)** loss of antigen processing and presentation machinery, including MHC I, β2-microglobulin (B2M), and transporter-associated antigen processing (TAP1/2), impairs T-cell–mediated cytotoxicity; **(3)** defects in IFN-γ signaling, such as JAK1/2 mutations or STAT1 inactivation, blunt immune activation and antigen presentation; (**4)** overexpression of inhibitory checkpoint ligands (PD-L1, galectin-9, CEACAM1, FGL1, CD155, CD112, among others) dampens T-cell function; and **(5)** activation of oncogenic pathways (PI3K/AKT/mTOR, WNT/β-catenin, MAPK) promotes immune exclusion and supports the survival of immunosuppressive cells.

**Figure 2.**
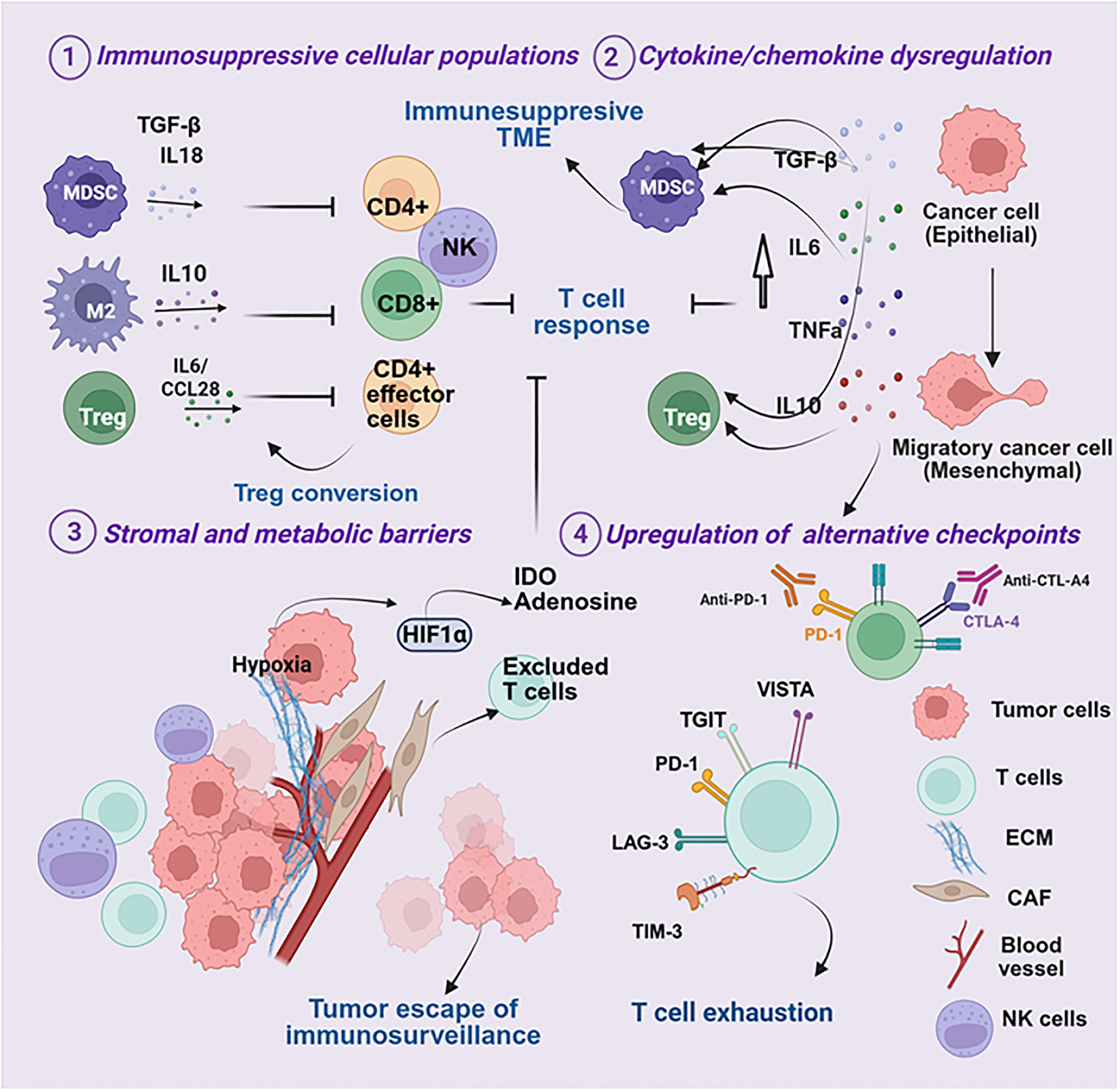
Tumor-extrinsic mechanisms of resistance to ICIs. **(1)** Recruitment of immunosuppressive cell populations (Tregs, MDSCs, and M2 macrophages) suppresses T-cell cytotoxicity and limits effective anti-tumor responses; **(2)** dysregulated cytokine and chemokine signaling (e.g., TGF-β, TNF-α, IL-6, IL-10) enhances immune suppression and reinforces T-cell dysfunction; **(3)** stromal and metabolic alterations—including hypoxia, abnormal vasculature, and extracellular matrix (ECM) remodeling—create physical barriers to immune cell infiltration and promote tumor immune escape; and **(4)** upregulation of alternative immune checkpoints (LAG-3, TIM-3, TIGIT, VISTA) drives T-cell exhaustion and limits response to PD-1/PD-L1 blockade.

Resistance to ICIs can be broadly divided into tumor-intrinsic and -extrinsic mechanisms. Intrinsic mechanisms arise from genetic and signaling alterations within tumor cells that impair immune recognition or effector function. Extrinsic mechanisms occur in the TME, where cellular and soluble factors create an immunosuppressive milieu. In practice, these mechanisms are highly interconnected and often overlap, collectively shaping the degree and durability of response [124,125] (Figures 1 and 2).

### 1. Tumor-intrinsic resistance mechanisms

#### Tumor mutation burden and neoantigen load:

One of the strongest correlates of ICI efficacy is TMB and the associated generation of non-synonymous mutations that produce immunogenic neoantigens [126]. High TMB, particularly observed in mismatch repair-deficient tumors, is associated with improved ICI responsiveness [127–129] (Figure 1.1). Melanoma exemplifies this phenomenon: ultraviolet-induced mutagenesis produces one of the highest TMBs among solid tumors, generating a rich neoantigen landscape that drives robust immune recognition [130,131]. Consistent with this concept, recent clinical analyses have shown that patients receiving biomarker-guided dual-matched therapies (combining targeted agents with ICIs) can experience durable clinical benefit, including long-term survival exceeding 1.5 years in some cases [132]. While the overall TMB in HNSCC is intermediate compared to melanoma, a significant subset of HNSCC patients have elevated TMB, and this is predictive of better responses to ICIs [133,134].

HPV status significantly shapes the neoantigen repertoire with HPV+ tumors, in addition to tumor-derived neoantigens, presenting viral antigens that enhance immune responses, whereas HPV- tumors often show neoantigen loss [135]. By contrast, TNBC exhibits lower TMB, limiting neoantigendriven immunity and contributing to modest ICI response rates [136]. Highly immunogenic tumors such as melanoma, HNSCC, and non-small cell lung carcinoma, with enriched TMB and neoantigen landscapes, are more responsive to ICIs than tumors with low TMB, such as TNBC, prostate, and pancreatic cancers. Consequently, low-TMB tumors often exhibit poor T cell infiltration and a “cold” tumor immune TME [131,137,138] (Figure 1.1). However, TMB is not universally predictive of ICI response [139]. Tumors with high neoantigen load may still develop resistance if these neoantigens are weakly immunogenic [140,141] or actively suppress immune responses (inhibitory neoantigens) [141–145]. Current strategies focus on radiotherapy, chemotherapy, and vaccines in combination with immunotherapy to increase neoantigen availability and enhance immunogenicity [146–148].

Recent evidence indicates that neoantigens can also arise from post-translational modifications, including glycosylation (O-linked β-N-acetylglucosamine), phosphorylation (phospho-neoantigens), and alternative RNA splicing [149–151]. These modifications expand antigenic diversity, create unique epitopes, and provide an immunological signature of the “transformed self” recognized by T cells [152,153]. Some phospho-neoantigens are shared across multiple tumor types and patients, offering the potential for immunotherapeutic targeting beyond personalized approaches [154].

#### Impaired antigen processing and presentation:

Defects in antigen presentation are a well-documented mechanism of ICI resistance [155] (Figure 1.2). Effective CD8^+^ T-cell recognition requires intact MHC-I-mediated antigen processing and presentation of tumor antigens [156]. Loss of MHC-I surface expression or structural disruption allows tumors to evade T-cell surveillance [157]. Mutations in β2-microglobulin (B2M) destabilize MHC-I complexes [158], while deficiencies in transporters associated with antigen processing (TAP1/2) or endoplasmic reticulum (ER) aminopeptidases (ERAP1/2) impair peptide translocation and loading [159,160].

These alterations occur across melanoma, HNSCC, and TNBC, with context-dependent contributions [161,162]. In melanoma, B2M mutations and MHC-I downregulation are strongly linked to acquired resistance after initial PD-1 blockade, and IFN-γ pathway defects further reduce antigen presentation [163,164] (Figure 1.2). In HNSCC, antigen presentation status is influenced by HPV status, with HPV+ tumors generally retaining intact MHC-I expression and an inflamed TME, whereas HPV- tumors more often lack MHC-I, correlating with poor ICI response [124]. Beyond MHC-I, impaired MHC-II presentation by tumor or myeloid cells attenuates CD4^+^ T-cell-mediated immunity, adding another layer of immune evasion [165]. In TNBC, B2M mutations are less common, with resistance often driven by transcriptional or epigenetic repression of antigen presentation machinery [161,166].

#### Disruption of IFN-γ signaling:

The IFN-γ pathway is central to tumor immune recognition (Figure 1.3), driving expression of MHC-I and immunoregulatory molecules such as PD-L1 via Janus kinases 1 and 2 (JAK1 and JAK2) and the signal transducer and activator of transcription 1 (STAT1) activation [167]. Disruption of this pathway by tumor-intrinsic alterations is a well-established mechanism of primary resistance to ICIs. Loss-of-function mutations or epigenetic silencing of JAK1/JAK2, and downstream transcriptional regulators impair the IFN-γ–mediated induction of the antigen presentation machinery and checkpoint ligands, enabling tumor immune evasion by rendering tumors “invisible” to cytotoxic T cells, even in the context of high TMB [164,168]. Consequently, in melanoma, HNSCC, and TNBC, JAK/STAT pathway inactivation prevents IFN-γ-induced upregulation of MHC-I and PD-L1, contributing to primary resistance despite an increased neoantigen burden [163,169] (Figure 1.3).

In HNSCC (particularly HPV+) and TNBC, preserved IFN-γ signaling drives strong PD-L1 induction (Figure. 1.3), limiting T-cell activity and promoting adaptive resistance [170]. HPV- and tobacco-associated tumors often harbor JAK/STAT defects, reducing IFN-γ signaling, diminishing antigen presentation, and promoting tumor immune evasion [171]. Amplification of negative regulators, such as suppressor of cytokine signaling 1 (SOCS1) and protein inhibitor of activated STAT4 (PIAS4), further suppresses IFN-γ signaling [169], thereby facilitating immune evasion.

Paradoxically, intact IFN-γ signaling can also drive adaptive resistance, as chronic exposure induces chronic PD-L1 expression, dampening T-cell activity and fostering immune evasion [170]. Thus, IFN-γ signaling exerts a dual influence: loss abrogates immune recognition and drives primary resistance, while persistent activation promotes adaptive resistance through PD-L1-mediated suppression.

#### Upregulation of immune checkpoint ligands by tumor cells:

Tumor cells evade immune pressure by upregulating several inhibitory checkpoint ligands on their surface, effectively suppressing activation of the T-cells expressing cognate receptors (Figure 1.4). The expression of such ligands within the TME can quench immune effector functions, promote regulatory or suppressive subsets of cells, and allow the tumor to evade immune attack [172]. This mechanism is central to the processes of immune escape and contributes to both primary and acquired resistance to ICI [173]. PD-L1 expression has been extensively studied as a predictive biomarker for response to PD-1/PD-L1 blockade across multiple cancer types, including melanoma, HNSCC, NSCLC, and TNBC [97,135,162,170,174]. Higher PD-L1 expression is generally associated with increased response rates to checkpoint inhibitors; however, substantial clinical benefit is also observed in PD-L1-negative tumors. This indicates that PD-L1 status is neither a sufficient nor necessary condition for therapeutic response [124,175]. The limitations of PD-L1 as a biomarker reflect tumor heterogeneity, emphasizing the need for additional predictive indicators beyond PD-L1 alone [174].

Beyond PD-L1, many tumors express ligands for other checkpoint receptors (Figure 1.4), for instance: LAG-3, T cell immunoglobulin and mucin-domain-containing-3 (TIM-3), T cell immunoreceptor with immunoglobulin and tyrosine-based inhibitory motif domain (TIGIT) and V-domain Ig suppressor of T cell activation (VISTA) [176,177]. TIM3 interacts with several ligands, including galectin-9, phosphatidylserine, carcinoembryonic antigen-related cell adhesion molecule 1 (CEACAM1), and high mobility group protein B1 (HMGB1), as well as HLA-B-associated transcript 3 (BAT3). LAG-3 binds fibrinogen-like protein 1 (FGL1), lectins galectin-3 (Gal-3), and lymph node sinusoidal endothelial cell C-type lectin (LSECtin). TIGIT interacts with CD155 (PVR) and CD112 (PVRL2) [178,179], expressed on tumor cells and competing with ligand-expressing APCs [180,181]. In melanoma [179] and HNSCC [182], the CD155/TIGIT axis is prominent, contributing to ICI resistance despite highly immune TME. TNBC cells express CD155 and CD112 as well, promoting TIGIT-mediated immunosuppression, which is linked to poor prognosis with anti-PD-1 therapy [183].

Tumor-intrinsic mechanisms can include PD-L1-enriched exosomes, which extend immunosuppression systemically by inhibiting T-cell activation, promoting apoptosis, and enhancing Treg function [184]. A reduction in exosomal PD-L1 during treatment correlates with improved responses, suggesting its potential as a liquid biopsy biomarker [184].

#### Oncogenic pathway activation:

Oncogenic signaling pathways play a central role in immune evasion and ICI resistance across melanoma, HNSCC, and TNBC. Activation of PI3K/AKT/mammalian target of rapamycin (mTOR), WNT/β-catenin, and mitogen-activated protein kinase (MAPK) pathways reshapes the TME and suppresses immune infiltration (Figure. 1.5) [185–188]. However, these pathways are often characterized by complex feedback loops and compensatory mechanisms that sustain tumor growth and contribute to immune escape [189–191]. Current efforts focus on optimizing drug combinations, dosing schedules, and patient selection to maximize therapeutic benefit. Rationally combining these pathway inhibitors with ICIs represents a promising approach to overcome immune exclusion and treatment resistance [192,193].

Aberrant PI3K/AKT/mTOR activation is common in TNBC and HNSCC, often resulting from PTEN loss or PI3K mutations [194,195]. This pathway supports tumor proliferation and immune escape by reducing CTL infiltration and enriching immunosuppressive myeloid populations [196]. Some PI3K/AKT/mTOR inhibitors have demonstrated promising preclinical activity in breast cancer [197,198], but their therapeutic efficacy has been partially limited by acquired resistance, as well as by substantial adverse effects [199]. Preclinical and clinical studies show that combining PI3K inhibitors with ICIs improves antitumor responses in melanoma and TNBC [200,201], with ongoing trials investigating similar strategies in HNSCC [24]. While pharmacological AKT inhibition showed no impressive effects, genetic silencing of all AKT paralogs triggered mTOR-dependent melanoma cell death, rescuable by kinase-active AKT1 [191]. A novel dual PI3K/mTOR inhibitor suppressed both proliferation and growth of MAPK inhibitorresistant melanoma *in vitro* and *in vivo*, showing promise as a well-tolerated therapy for frontline and resistant disease [202,203]. Key strategies also include leveraging pan-PI3K inhibitors for broader pathway targeting in HNSCC, as well as incorporating epigenetic modifiers such as histone deacetylase inhibitors (HDACi) or DNA methyltransferase inhibitors to disrupt alternative signaling routes and overcome compensatory resistance mechanisms [195,196].

Aberrant WNT/β-catenin signaling is not only a key mechanism of tumorigenesis but a significant modulator of TME, contributing to immune exclusion and resistance to ICIs across several cancers [190,204]. This phenomenon has been extensively demonstrated in melanoma and is gaining recognition in HNSCC and TNBC [42,205]. In preclinical models and clinical settings, the WNT/β-catenin pathway prevents dendritic cell and T cell infiltration, generating a “cold” immune TME and driving resistance to PD-1/CTLA-4 blockade [41] (Figure 1.5). Mechanistically, β-catenin activation suppresses CCL4, impairing the recruitment of CD103+ dendritic cells essential for CD8^+^ T-cell priming [188]. The WNT/β-catenin pathway contributes to the preservation or expansion of Tregs via IL-10 release, thereby reinforcing an immunosuppressive TME [206]. In TNBC, characterized by a generally “cold” immune landscape, the WNT/β-catenin pathway’s influence on immune exclusion is significant, making it an essential target for therapeutic intervention. Furthermore, the interplay between the WNT/β-catenin pathway and other metabolic pathways, such as those involving IDO and adenosine, can further solidify an immunosuppressive microenvironment, presenting additional challenges for immune cell function in the face of ICI treatment [207].

Mutations in the MAPK pathway (e.g., BRAF-V600E in melanoma, diverse mutations in HNSCC) contribute to ICI resistance via cytokine induction, diminished antigen presentation, and expansion of regulatory cells [192,208]. In melanoma, acquired resistance to MAPK-targeted therapy is associated with decreased MHC-I expression, reduced T-cell infiltration, and diminished immunotherapy efficacy through IL-6/IL-10-mediated suppression and Treg expansion [209,210]. In contrast, HNSCC displays a more nuanced signaling context, where some MAPK-mutant tumors exhibit better CD8^+^ T-cell infiltration and improved ICI outcomes. In TNBC, MAPK dysregulation similarly contributes to immune escape, limiting responses to combination therapy [211].

### 2. Tumor-extrinsic resistance mechanisms

The TME is highly heterogeneous, consisting of malignant cells, immune populations, stromal elements, vasculature, and extracellular matrix. Emerging evidence highlights the complex crosstalk among these components critically shaping immunosuppression, remodeling anti-tumor immune responses, and dictating therapeutic sensitivity [25,212]. Resistance mechanisms within the TME arise from both cellular and non-cellular factors that suppress local immunity, including expansion of inhibitory immune populations, physical exclusion of effector T cells, and metabolic constraints that induce T-cell exhaustion [213] (Figure 2).

#### Immunosuppressive cell populations:

Resistance across solid tumors is reinforced by immunosuppressive subsets such as Tregs, MDSCs, and M2-polarized tumor-associated macrophages (TAMs), which are key mediators in melanoma, HNSCC, and TNBC (Figure 2.1). These cells inhibit cytotoxic T-cell activity by secreting IL-10, transforming growth factor-beta (TGF-β), and other suppressive molecules [214,215]. In melanoma, tumors arise within a “hot” immune milieu enriched in CD8^+^ T cells [216] (Figure 2.1). However, abundant Tregs blunt T cell cytotoxic activity, enforce tolerance, and promote therapy resistance. In HNSCC, the immune landscape is shaped by etiological diversity. HPV+ tumors display dense T-cell infiltration with frequent Tregs and stromal activation [217], whereas tobacco-associated HNSCC exhibits immune desertification and poor ICI responses [109,171]. Across both HPV+ and HPV- tumors, cytotoxic CD56^dim^ NK cells, though abundant, are suppressed by KIR signaling [111]. HPV+ tumors also exploit E6/E7 oncoproteins to impair antigen presentation, dampen NK activity, and reprogram cytokine signaling [13,218]. In contrast, TNBC is often immunologically “cold” and characterized by scarce CTLs and an enrichment with immunosuppressive MDSCs and M2-polarized macrophages, which may limit ICI responsiveness [219]. Within these tumor contexts, several immunosuppressive cell populations are central drivers of immunotherapy resistance [214,215].

#### Cytokine/chemokine dysregulation:

Cytokine dysregulation in the TME is typically initiated by oncogenic signaling, hypoxia-induced stress responses, and innate immune activation, which together establish self-sustaining cytokine loops that shape an immunosuppressive microenvironment (Figure 2.2). IL-6 drives MDSC expansion via STAT3 and IDO signaling [220], skewing T cell differentiation toward Th17 phenotypes [221]. Tumor necrosis factor-alpha (TNF-α) signaling, despite activation of CTL, enhances MDSC-mediated immunosuppression by promoting the survival and suppressive function of these cells [222]. TGF-β reprograms immune and stromal metabolism, promoting epithelial-to-mesenchymal transition (EMT) [223]. Together, IL-6, TNF-α, and TGF-β drive T-cell exhaustion, enhance the expression of PD-1 and CTLA-4, expand Tregs, and impair NK activity [221,223,224]. These cells subsequently release inhibitory mediators that block effector T-cell infiltration into the tumor, establishing a suppressive niche and fostering immune exclusion [225,226].

#### Stromal and metabolic barriers:

Spatial heterogeneity across melanoma, HNSCC, and TNBC creates barriers to immune infiltration and ICI efficacy (Figure 2.3). Beyond purely “hot” or “cold” classifications, many solid tumors exhibit immune-excluded phenotypes, characterized by immune cells—especially CD8^+^ T cells—localized to the tumor periphery or stroma but unable to penetrate the tumor parenchyma [227,228]. This spatial immune segregation, frequently driven by dense extracellular matrix (ECM) deposition, cancer-associated fibroblast (CAF) activation, and TGF-β–mediated signaling, creates physical and biochemical barriers that prevent effective cytotoxic engagement [226].

Such immune-excluded environments are especially prominent in TNBC, where stromal TGF-β signaling and myofibroblast expansion contribute to peripheral T-cell trapping and therapeutic resistance [229]. Similarly, subsets of HNSCC show collagen crosslinking and stromal niche formation driven by CAFs, contributing to immune exclusion [230]. Together, these features underscore that stromal remodeling, not only cellular immunosuppression, represents a complementary axis of immune evasion across solid tumors.

Dense desmoplastic stroma, enriched in CAFs, collagen, and hyaluronan, further restricts T-cell infiltration in HNSCC and TNBC [231,232]. CAF-derived IL-6 and JAK2/STAT3 activation promote fibroblast proliferation, Th17 polarization, and immunosuppressive cytokine release [218,233]. In melanoma, resistance is compounded by metabolic rewiring, including activation of indoleamine 2,3-dioxygenase (IDO) and adenosine accumulation, which suppresses T- and NK-cell function and blunts PD-1 blockade efficacy [231,233,234].

Epithelial–mesenchymal transition (EMT) remodeling further strengthens stromal barriers (Figure 2.3), particularly in HNSCC and TNBC, where TGF-β, IL-6, Wnt, Notch, and hypoxia pathways collectively drive immune exclusion and therapeutic resistance [235,236]. Hypoxia, a common feature across all three cancers, stabilizes hypoxia-inducible factor-1α (HIF-1α), thereby promoting angiogenesis, PD-L1 expression, recruitment of suppressive cells, and activation of metabolic checkpoints such as IDO and adenosine [237–239] (Figure 2.3).

HNSCC and TNBC are particularly hypoxic due to dense stroma and high metabolic demand, whereas melanoma harbors localized hypoxic niches driving immune escape [240–242]. In HNSCC, hypoxia is particularly pronounced due to the high metabolic demand of rapidly proliferating tumor cells and extensive stromal fibrosis. Hypoxia in HNSCC promotes angiogenesis through vascular endothelial growth factor (VEGF), induces PD-L1 upregulation, and facilitates recruitment of MDSCs and TAMs, thereby driving tumor immune evasion [233]. In TNBC, elevated HIF-1α enhances VEGF secretion and stromal fibrosis, fostering metastasis and resistance [243]. Accumulation of lactate and adenosine under hypoxic stress also suppresses T and NK cell function, limiting ICI efficacy [244]. In melanoma, although global hypoxia is less pronounced, localized niches activate CCL28 and CXCL12, attracting Tregs and MDSCs and reinforcing immune suppression [245]. Collectively, stromal remodeling, metabolic reprogramming, and hypoxia form an interlinked network that shapes the TME, restricts immune infiltration, and drives resistance to ICI therapies across melanoma, HNSCC, and TNBC.

#### Alternative immune checkpoints:

Beyond PD-1 and CTLA-4, several alternative inhibitory pathways—including TIM-3, LAG-3, TIGIT, and VISTA—contribute to sustained immune exhaustion and tumor immune evasion [172,246]. These pathways signal through unique mechanisms to suppress T cell proliferation and cytokine production, fostering a state of chronic exhaustion and reduced cytotoxic activity. The engagement of these alternative checkpoints as compensatory mechanisms in response to ICI therapy underscores the development of secondary acquired resistance.

TIM-3, often co-expressed with PD-1 on T cells, NK cells, and Tregs, suppresses effector functions and is associated with poor survival [247,248]. Dual blockade of PD-1 and TIM-3 has shown potential to restore T-cell activity [249].

LAG-3 is widely expressed on activated and exhausted T cells, NK cells, B cells, and plasmacytoid dendritic cells. It synergizes with PD-1—particularly in melanoma and HNSCC—leading to profound T-cell exhaustion and resistance to anti-PD-1/PD-L1 therapy. In TNBC, LAG-3 expression demonstrates a context-dependent role, but combined targeting of PD-1 and LAG-3 offers promise for overcoming immunosuppression [181,250].

TIGIT interacts with CD155 or CD112 to suppress CTL and NK cell activity, enhancing IL-10 secretion and Treg expansion [251]. High TIGIT levels predict resistance in melanoma and TNBC, and TIGIT inhibition can enhance responses to PD-1 blockade [180].

VISTA, expressed on myeloid cells and T cells, dampens T-cell activation and promotes immunotherapy resistance, particularly in inflamed tumors such as HNSCC and TNBC [252,253]. Collectively, these alternative checkpoints interact with cytokine networks and stromal barriers, establishing a multifaceted immunosuppressive tumor microenvironment. Their cooperative roles support clinical investigation of dual or triple checkpoint blockade strategies to overcome resistance [177,249].

### 3. Systemic mechanisms

Resistance to ICIs is shaped by systemic host determinants [19,20,254]. Host-related factors, including chronic inflammation, nutritional and metabolic status, and the microbiome, modulate both intrinsic (mutational landscape, cytokine signaling, and metabolism) and tumor extrinsic mechanisms (immune cell trafficking and effector function within the TME) [233,255]. These systemic influences differ in relative importance across melanoma, HNSCC, and TNBC, yet collectively define immune competence, treatment tolerance, and the durability of anti-tumor responses.

#### Chronic inflammation and comorbidities:

Systemic inflammation, driven by smoking, alcohol use, obesity, chronic infections, and aging, impairs antigen presentation, blunts T-cell priming, accelerates immune senescence, and thereby reduces ICI efficacy [256]. In melanoma, chronic ultraviolet-driven inflammation and age-related immune-senescence reduce naive T-cell pools and cytokine fitness, limiting response durability in older or frail patients. Body-composition metrics in melanoma patients further forecast ICI outcomes: low skeletal muscle index, high subcutaneous adipose tissue density, and sarcopenia correlate with inferior progression-free and overall survival on ICI therapy. Conversely, better pre-diagnosis diet quality (e.g., higher Healthy Eating Index) has been linked to thinner primary tumors at presentation, underscoring the role of modifiable host determinants in shaping immunity.

In HNSCC, tobacco and alcohol exposure drive inflammation, myeloid skewing, and frailty, all of which correlate with inferior ICI outcomes [257]. HPV+ HNSCC is generally more immunogenic, yet systemic comorbidities and malnutrition remain detrimental. For instance, cachexia and sarcopenia impose substantial metabolic stress that impairs T-cell priming and effector cytokine production [258,259]. Dysphagia, mucositis, and treatment-related catabolism frequently result in weight loss and immune dysfunction. Validated tools such as patient-generated subjective global assessment (PG-SGA) link poor nutritional status to advanced stage and worse survival; structured interventions (dietary counseling, prophylactic feeding tubes) mitigate severe toxicities and preserve immune competence during chemoradiation [260].

In TNBC, systemic inflammation is often linked to obesity, insulin resistance, and adipokine dysregulation (elevated leptin, reduced adiponectin), which increase circulating IL-6 and TNF-α, promote myelopoiesis, and skew toward suppressive myeloid phenotypes [261,262]. Systemic metabolism strongly influences TNBC aggressiveness. Preclinical studies show that Western-style diets accelerate tumor growth and blunt chemotherapy, whereas fasting-mimicking or ketogenic diets enhance immune fitness and prolong survival in murine TNBC [263]. Clinically, lower circulating glucose has been associated with improved outcomes in some TNBC cohorts [264], consistent with tumor–immune metabolic competition. Nutritional and metabolic interventions are now being evaluated as adjunctive strategies to potentiate ICI efficacy [260].

#### Microbiome dysbiosis:

Melanoma and HNSCC are uniquely shaped by their interaction with microbiota, given their interface with heavily colonized barrier surfaces—the skin and oral cavity, respectively—which profoundly influence tumor-immune dynamics. The distinct microbial ecology at these sites plays a pivotal role in local immunity [265]. Microbial metabolites, particularly short-chain fatty acids, modulate chemokine production (CCL5, CXCL10), enhance T-cell metabolism, and promote intra-tumoral trafficking [266]. In contrast, dysbiosis impairs antigen presentation, reduces CD8+ T cell activation, and promotes expansion of Tregs, creating an environment conducive to immune evasion [267].

In melanoma, multiple studies demonstrate that fecal microbiota transplantation (FMT) from ICI responders into non-responders restores intra-tumoral immune infiltration and improves clinical outcomes, with OSR approaching 65%, including 20% complete responses [268–270]. In HNSCC, the oral microbiome exerts both cancer- and therapy-relevant effects [271,272]. In resected HNSCC patients, a shift toward health-associated taxa (e.g., *Streptococcus*, *Rothia*) and away from *Capnocytophaga*, *Prevotella*, and *Leptotrichia* correlated with improved three-year disease-specific survival [272,273]. However, direct evidence linking oral or gut microbiome modulation to ICI efficacy in HNSCC remains limited and warrants prospective studies [274]. In TNBC, baseline gut microbial diversity correlates with longer PFS in patients treated with atezolizumab plus chemotherapy [275]. Preclinical TNBC models further suggest that restoring beneficial microbial metabolites, such as branched-chain amino acids, enhances PD-1-mediated immunity [276]. Collectively, the microbiome functions both as a biomarker of ICI benefit and as a modifiable co-therapeutic target across melanoma, HNSCC, and advanced TNBC.

## Conclusions and Lessons from a Decade of Forefront Therapy

A decade of clinical experience with ICI has fundamentally shifted the therapeutic paradigm in cancer, particularly for melanoma, HNSCC, and TNBC. These advances have redefined survival outcomes and established immunotherapy as a core pillar of modern oncology. However, the management of immune-related adverse events (irAEs) remains a critical challenge [277]. irAEs can affect virtually any organ system—most commonly the skin, gastrointestinal tract, liver, and endocrine glands—and range from mild to life-threatening [278]. Severe, multi-organ toxicities limit the broader application of ICIs, particularly in frail or comorbid patients. Several prospective clinical strategies are under active investigation—notably IL-6/IL-6R blockade (e.g., tocilizumab) and TNF-α or gut-selective agents (infliximab, vedolizumab) for severe or steroid-refractory irAEs, as well as targeted approaches such as abatacept, JAK inhibitors, IL-1 blockade, and microbiome modulation—which aim to reduce toxicity severity and dependence on high-dose corticosteroids while maintaining efficacy [221–226]. Early clinical signals are encouraging, but larger randomized studies with survival and quality-of-life endpoints are required to establish standard mitigation strategies [279].

Recent clinical and translational research increasingly emphasizes combination strategies that concurrently target multiple resistance mechanisms (Tables 2 and 3). Key developments include dual checkpoint blockade, targeting PD-1 together with LAG-3 [280], TIGIT [281], or other inhibitory receptors [282], which has demonstrated efficacy in treating refractory tumors. Neoadjuvant immunotherapy, involving administration of ICIs before surgical intervention, has shown improved pathological responses and survival outcomes in melanoma and TNBC, offering insight into early tumor–immune dynamics [50,283].

Novel immunomodulatory agents such as vidutolimod (TLR9 agonist) [284] and WTX-124 (tumor-activated IL-2 prodrug) [285] are designed to enhance both innate and adaptive immunity while minimizing systemic toxicity. Adoptive T-cell therapies, particularly TIL products such as lifileucel (Amtagvi^™^), along with personalized cancer vaccines, offer promising avenues for patients with ICI-refractory disease [286–288]. Similarly, antibody–drug conjugates (ADCs) such as sacituzumab govitecan, a Trop-2-directed ADC, have shown substantial benefit in heavily pretreated TNBC [289]. These targeted therapies address tumor-specific vulnerabilities and contribute to a multi-modal framework for overcoming tumor heterogeneity and resistance.

In addition to these approaches, next-generation immunotherapies are being developed to further enhance clinical outcomes [290]. Oncolytic viruses represent a promising avenue for overcoming resistance to ICIs. The FDA-approved talimogene laherparepvec (T-VEC), a genetically modified herpes simplex virus, has demonstrated durable responses and improved overall survival in advanced melanoma by promoting tumor lysis and systemic immune activation [302]. Ongoing trials are evaluating T-VEC in combination with ICIs and radiotherapy to further augment antitumor immunity [303]. In HNSCC, clinical studies are investigating intratumoral viral delivery and combination regimens, particularly in HPV-positive or immunologically “cold” tumors where viral priming may enhance immune infiltration [304]. For TNBC, oncolytic HSV and reoviruses are under clinical evaluation for their ability to induce immunogenic cell death and reshape the TME [305].

Epigenetic reprogramming provides an additional avenue to overcome immune resistance. In melanoma, inhibitors of DNA methyltransferases (DNMTs) and HDACs can restore antigen presentation, reactivate silenced immune genes, and enhance checkpoint blockade efficacy [306,307]. In HNSCC and TNBC, epigenetic therapies are under investigation to reverse tumor-induced immunosuppression and restore effective immune signaling, potentially sensitizing tumors to chemotherapy and ICIs [308,309]. Moreover, targeting DNA damage response pathways has emerged as a complementary approach. PARP inhibitors, such as Olaparib, have been approved for BRCA-mutated TNBC [310], with ongoing trials exploring combinations with ICIs, AKT inhibitors, and ADC [311–313].

The integration of these next-generation therapies with current ICIs underscores a dynamic shift towards personalized cancer treatments. Strategies such as optimizing nutrition, preserving metabolic and immune fitness, and modulating the microbiome through dietary fiber, pre/probiotics, or FMT are being investigated as adjunctive therapies to enhance immune competence, reduce toxicities, and extend the durability of checkpoint blockade [266,314]. Recent progress in single-cell transcriptomics, spatial multi-omics, and AI-assisted response prediction is also refining patient selection and guiding personalized combinations [315–318].

Collectively, these developments highlight a translational shift toward precision immunotherapy—integrating genomic, metabolic, and immunologic profiling to tailor treatments while mitigating toxicity. Lessons from the past decade underscore that durable benefit from ICIs requires not only overcoming resistance but also mastering the balance between immune activation and immune tolerance, ensuring that the next generation of immunotherapies achieves maximal efficacy with minimal harm.

## Figures and Tables

**Table 1.** Shared and cancer-specific features shaping ICI response in melanoma, HNSCC, and TNBC.

	Melanoma	HNSCC	TNBC
**Common resistance mechanisms**	Impaired antigen presentation (B2M, MHCI mutations) Defects in the IFN-γ signaling pathway (JAK1/2 mutations) Activated oncogenic pathways Suppressive TME—enriched in Treg, MDSCs, and TAMs		
**Unique cancer features**	↑ UV TMB and neoantigen load ↑ Melanocyte lineage antigens ↑ Immune infiltration	HPV+ (E6/E7) and HPV- subtypes Tobacco-related mutations and neoantigen landscape Unique TME (↑NK-cell infiltration (CD56dim)	↓ TMB Poor antigen presentation Strong stromal barriers ↑ Immune “cold”TME
**Response to ICI monotherapy**	~40–45% ORR	~15–20% ORR	~5–20% ORR

**Table 2.** Key clinical trials establishing ICIs as a standard of care in melanoma, HNSCC, and TNBC.

Trial Number	Treatment	Subject	Reference
***Melanoma***			
NCT03396952	Pembrolizumab + Ipilimumab + High-dose Aspirin	Advanced Metastatic Melanoma	[47]
NCT01844505	Nivolumab or Nivolumab + Ipilimumab vs. Ipilimumab Alone	Advanced Melanoma	[48]
NCT03470922	Relatlimab + Nivolumab vs. Nivolumab Alone	Advanced Melanoma	[49]
NCT04949113	Neoadjuvant Ipilimumab + Nivolumab vs. Standard Adjuvant Nivolumab	Stage III Melanoma	[50]
NCT04274816	Intradermal Tremelimumab (low dose)	Early-stage Melanoma (Stage I–II)	[51]
NCT01866319 (KEYNOTE-006)	Pembrolizumab vs. Ipilimumab	Metastatic Melanoma	[31]
NCT00323206	Intratumoral IL-12 plasmid + electroporation	Metastatic Melanoma (Phase I dose escalation)	[52]
NCT02275416	UV1 peptide vaccine + Ipilimumab	Unresectable Metastatic Melanoma	[53]
NCT02752074 (ECHO-301/KEYNOTE-252)	Epacadostat + Pembrolizumab vs. Pembrolizumab alone	Unresectable/Metastatic Melanoma	[54]
NCT02475213	Enoblituzumab + Pembrolizumab	Advanced Solid Tumors (including Melanoma)	[55]
NCT03693612 (INDUCE-2)	Feladilimab + Tremelimumab	Advanced Solid Tumors (including Melanoma)	[56]
NCT03776136 (LEAP-004)	Lenvatinib + Pembrolizumab	Unresectable Stage III/IV Melanoma with progression on prior PD-1/PD-L1 therapy	[57]
NCT00179608	Lenalidomide + Dacarbazine	Chemo-naïve Metastatic Melanoma patients	[58]
NCT00864253	Nab-paclitaxel vs. Dacarbazine	Metastatic Melanoma	[59]
NCT03178851	Cobimetinib + Atezolizumab	BRAF V600 WT Advanced Melanoma, post–PD-1 therapy	[60]
NCT00086489	Tremelimumab	Advanced Melanoma	[61]
NCT01656642	Atezolizumab + Vemurafenib ± Cobimetinib	Metastatic Melanoma (BRAF V600–mutant)	[62]
NCT00616564	ch14.18 + R24 antibodies combined with IL-2	Metastatic Melanoma (23 patients) and Sarcoma (4 patients)	[63]
NCT00631072	Autologous iNKT cell infusion	Stage IIIB–IV Melanoma	[64]
NCT04551352	TYRP1-TCB (RO7293583) — bispecific antibody targeting TYRP1 + CD3	Metastatic Melanoma (cutaneous, uveal, mucosal; TYRP1-positive)	[65]
***Head and Neck Cancers***			
KEYNOTE-048	Pembrolizumab mono; Pembro + chemo + 5-FU Cetuximab + chemo + 5-FU	Recurrent/Metastatic HNSCC	[66]
NCT02741570	Nivolumab + ipilimumab vs. EXTREME regimen	Recurrent/Metastatic HNSCC	[67]
NCT02252042	Pembrolizumab vs. methotrexate, docetaxel or cetuximab	Recurrent/Metastatic HNSCC	[68]
NCT03342911	Nivolumab + carboplatin + paclitaxel	Stage III-IV HNSCC	[69]
NCT04282109	Nivolumab + paclitaxel	Recurrent/Metastatic HNSCC	[70]
NCT02179918	PF-05082566 + pembrolizumab (anti-PD-1)	Advanced Solid Tumors	[71]
NCT02110082	Urelumab (4–1BB agonist) and cetuximab	Advanced/Metastatic HNSCC	[72]
***Triple Negative Breast Cancer***			
NCT02622074	Pembrolizumab + Chemotherapy as Neoadjuvant	Early-Stage TNBC	[73,74]
NCT04613674	Camrelizumab + Chemotherapy vs. placebo + chemotherapy	Early or Locally Advanced TNBC	[75]
NCT03289819	Neoadjuvant Pembrolizumab/Nab-Paclitaxel Followed by Pembrolizumab/Epirubicin/Cyclophosphamide	Early-Stage TNBC	[76]
NCT02819518	Pembrolizumab combinations vs. Placebo + Chemotherapy	Previously untreated locally recurrent Metastatic TNBC	[77,78]
NCT03487666	Nivolumab and Capecitabine combined vs. alone	TNBC	[79]
NCT03125902	Atezolizumab + Paclitaxel vs. Atezolizumab Placebo + Paclitaxel	Previously Untreated Inoperable Locally Advanced or Metastatic TNBC	[80]
NCT02413320	Carboplatin + Paclitaxel then Doxorubicin + Cyclophosphamide vs. Carboplatin + Docetaxel	Stage I-III TNBC	[81]
NCT02447003	Pembrolizumab	Metastatic TNBC	[82]
NCT01375842	Atezolizumab	Metastatic TNBC	[83]
NCT01772004	Avelumab	Metastatic TNBC	[84]
NCT02657889	Pembrolizumab + Niraparib	Advanced/Metastatic TNBC	[85]
NCT03330405	ICIs + Avelumab + Talazoparib	Advanced TNBC	[86]
NCT02555657	Pembrolizumab vs. TPCe	Metastatic TNBC	[87]
NCT02734004	Olaparib + Durvalumab	Metastatic TNBC	[88]
NCT01042379	Paclitaxel with or without Pembrolizumab + adjuvant chemotherapy	Early-stage TNBC	[89]
NCT01633970	Nab-paclitaxel + Atezolizumab	Metastatic TNBC	[90]
NCT04129996	Angiogenesis inhibitor + Camrelizumab + Chemotherapy	Advanced immunomodulatory TNBC patients	[91]
NCT02425891	Nabpaclitaxel + Atezolizumab/Placebo	Metastatic TNBC	[92]
NCT02299999	Durvalumab vs. Chemotherapy	Metastatic TNBC	[93]

Trials are categorized by cancer type, treatment regimen, patient population, and corresponding reference. Included studies highlight both monotherapy and combination strategies that have shaped current clinical practice and informed emerging approaches to overcome resistance.

**Table 3.** Anti-PD-1 combination strategies with immune-stimulating agents to combat resistance to ICI.

Target/Agent	Mechanism of Action	Clinical Trial Phase Status	Tumor Type(s)	Key Outcome	References
EGFR inhibitors	Inhibition of EGFR promotes antigen presentation and enhances immune response to tumor cells	Phase II/III	HPV-related cancers	Improved ORR and overall survival in combination with PD-1 inhibitors	[68,291]
STAT3 inhibitors	Blockade of STAT3 suppresses an immunosuppressive transcription factor	Phase I	Advanced Solid Tumors	Tolerable safety profile; preliminary anti-tumor activity	[292]
CXCR2 inhibitors	Blockade of the chemokine receptor CXCR2 (primarily binds IL-8) reduces neutrophil recruitment	Phase I/II	Solid tumors	Combination with durvalumab did not improve ORR and had high adverse event rates.	[293]
IDO1 inhibitors	Blockade reduces immunosuppressive tryptophan metabolism and restores T and NK cell proliferation.	Phase I–III	Melanoma, Solid Tumors	Epacadostat + pembrolizumab: safe but failed phase III melanoma trial. Navoximod + atezolizumab: phase I ongoing	[207]
NKG2A inhibitors	Blockade restores CD8^+^ T cell and NK cell function.	Phase II	HNSCC	Combination with durvalumab + SOC did not improve PFS; results are pending.	[294]
B7-H3 inhibitors	Blockade enhances CD8^+^ T cell-mediated anti-tumor activity.	Phase II/III	Solid Tumors	Combination with retifanlimab improved ORR; acceptable safety profile.	[55]
VEGF inhibitors	Blockade of VEGF signaling reduces tumor angiogenesis and alleviates hypoxia-driven immunosuppression.	Phase III	Endometrial, Renal, Solid Tumors	Lenvatinib + pembrolizumab did not improve survival outcomes [126,127].	[295,296]
OX40 agonists	Costimulatory receptor activation enhances T cell proliferation and survival.	Phase I/II	Solid Tumors	Well tolerated; modest activity as monotherapy; combination trials with PD-1 ongoing.	[297]
4–1BB (CD137) agonists	Costimulatory receptor activation enhances T and NK cell activation.	Phase I/II	Solid Tumors	Some clinical activity; hepatotoxicity limited development; newer agents in trials with PD-1 inhibitors.	[298]
TLR agonists	Toll-like receptor activation stimulates innate immunity and dendritic cell function.	Phase I/II	Melanoma, HNSCC	Early signals of efficacy in combination with PD-1 inhibitors.	[299]
STING agonists	Activates STING pathway, inducing type I interferons and innate immune activation.	Phase I	Solid Tumors, Lymphoma	Safe but limited responses as monotherapy; PD-1 combinations under investigation.	[300]
CSF1R inhibitors	Reprograms tumor-associated macrophages from immunosuppressive to pro-inflammatory phenotype.	Phase I/II	Pancreatic and Solid Tumors	Combination with nivolumab showed limited clinical activity.	[301]

This table summarizes investigational approaches combining PD-1 inhibitors with other immune-modulating therapies. Each entry highlights the molecular target, its immunological mechanism of action, and the clinical outcome reported to date, ranging from early-phase safety and efficacy signals to negative or inconclusive phase II results.
